# Endoluminal Motion Recognition of a Magnetically-Guided Capsule Endoscope Based on Capsule-Tissue Interaction Force

**DOI:** 10.3390/s21072395

**Published:** 2021-03-30

**Authors:** Peisen Zhang, Jing Li, Weimin Zhang, Yang Hao, Gastone Ciuti, Tatsuo Arai, Paolo Dario, Qiang Huang

**Affiliations:** 1 Intelligent Robotics Institute, School of Mechatronical Engineering, Beijing Institute of Technology, Beijing 100081, China; 3120160106@bit.edu.cn (P.Z.); 3120150088@bit.edu.cn (Y.H.); 2 School of Electrical and Information Engineering, Beijing University of Civil Engineering and Architecture, Beijing 100081, China; 10902016@bit.edu.cn; 3 Beijing Advanced Innovation Center for Intelligent Robots and Systems, Beijing Institute of Technology, Beijing 100081, China; gastone.ciuti@santannapisa.it (G.C.); tarai118@jcom.zaq.ne.jp (T.A.); paolo.dario@santannapisa.it (P.D.); qhuang@bit.edu.cn (Q.H.); 4 The Biorobotics Institute, Scuola Superiore Sant’Anna, 56025 Pisa, Italy; 5 Key Laboratory of Biomimetic Robots and Systems, Beijing Institute of Technology, Ministry of Education, Beijing 100081, China

**Keywords:** robotic colonoscopy, magnetically-guided capsule endoscope, flexible force sensors, motion recognition

## Abstract

A magnetically-guided capsule endoscope, embedding flexible force sensors, is designed to measure the capsule-tissue interaction force. The flexible force sensor is composed of eight force-sensitive elements surrounding the internal permanent magnet (IPM). The control of interaction force acting on the intestinal wall can reduce patient’s discomfort and maintain the magnetic coupling between the external permanent magnet (EPM) and the IPM during capsule navigation. A flexible force sensor can achieve this control. In particular, by analyzing the signals of the force sensitive elements, we propose a method to recognize the status of the motion of the magnetic capsule, and provide corresponding formulas to evaluate whether the magnetic capsule follows the motion of the external driving magnet. Accuracy of the motion recognition in Ex Vivo tests reached 94% when the EPM was translated along the longitudinal axis. In addition, a method is proposed to realign the EPM and the IPM before the loss of their magnetic coupling. Its translational error, rotational error, and runtime are 7.04 ± 0.71 mm, 3.13 ± 0.47${}^{\circ }$, and 11.4 ± 0.39 s, respectively. Finally, a control strategy is proposed to prevent the magnetic capsule endoscope from losing control during the magnetically-guided capsule colonoscopy.

## 1. Introduction

**Figure 1:**
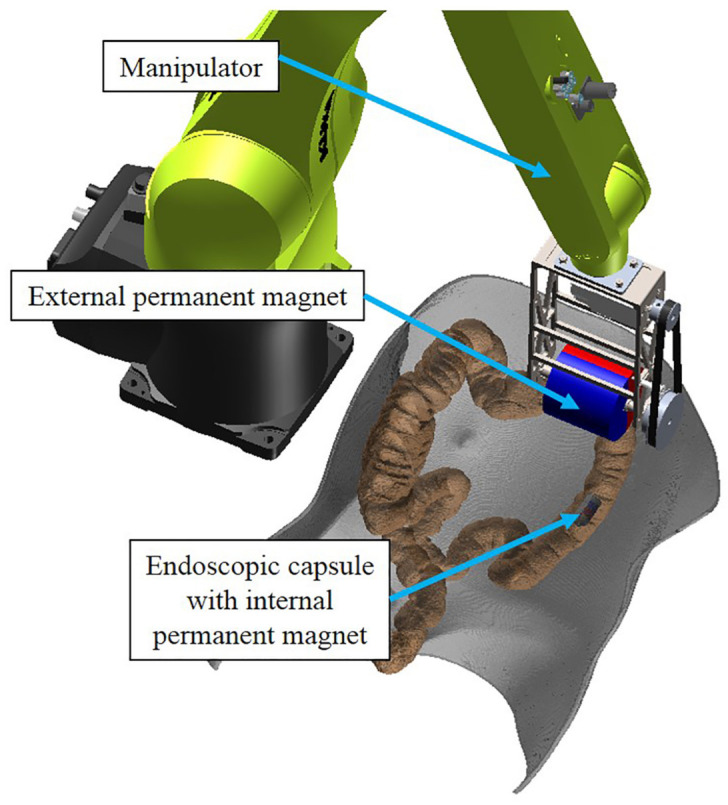
Generic working scenario of the magnetically-guided capsule colonoscopy.

Colorectal cancer (CRC) has caused more than 880 thousand deaths in 2018, ranking the second most deadly and third most common cancer worldwide [1,2]. Statistics indicate that advances in CRC screenings have reduced mortality in developed countries, even in the face of increased incidence [3,4,5]. Magnetically-guided capsule colonoscopy is one of the most promising technologies for the screening of large intestine diseases [6]. Its generic working scenario is illustrated in Figure 1. An external permanent magnet is installed on the end-effector of a robotic manipulator, and an internal permanent magnet is embedded inside the endoscopic capsule. Magnetic interactions between the magnetic sources can transmit motion free from physical barriers. By controlling the robotic manipulator, the external permanent magnet cannavigate the capsule along the colon. Compared with conventional endoscopes for which the driving force is applied from outside the anus through a tube with considerable stiffness, the magnetically-guided capsule endoscope causes less discomfort and pain for the patient, since its driving force is applied on the front-end of the magnetic capsule endoscope instead of pushing the endoscope from outside [7].

Numerous studies have been presented on topics of great interest in the development of magnetically-guided robotic capsule endoscopy, such as magnetic localization [8,9], magnetic interactions [10,11], drug delivery [12,13], and human–machine interface [14,15]. Various sensors, including Hall-effect sensor [16,17], inertial measurement unit (IMU) [18,19] and micro ultrasound transducer [20] are installed inside the magnetic capsule to gain its motion status under the limitations of physical barriers and line of sight. Many robotic capsule endoscopes used for large intestine detection has been developed, as shown in Table 1.

Since the magnetic capsule moves inside the human colon and the driving force is applied on the front of the capsule, the capsule-tissue interaction force should be monitored in real-time to avoid patient discomfort and intestinal injury. Analytical magnetic models, including dipole model [25,26], charge model [27] and current model [28], can be used to calculate the magnetic interaction force between EPM and IPM [29]. Using the magnetic localization method, IMU, and dipole model, researchers can control the magnetic force along the opposite direction of gravity, and use a control strategy that, by counteracting gravity, achieves magnetic levitation of the capsule endoscope [24]. They attain 19.5% of contact with the colon wall in experiments performed on a colonoscope training simulator. Considering the complex working environment of the capsule endoscope inside the human large intestine, the force sensor is still the best way to obtain accurate capsule-tissue interaction force information.

Flexible force-sensitive sensors are thin film pressure sensors, constructed by piezoresistive sensor array on flexible printed circuit board. Each sensor element responds to normal force by exhibiting a change in the through-film resistance [30]. Such sensors are widely used in electronic skin [31,32], touch-on flexible displays [33,34] and medical equipment [35,36] to measure the contact force directly acting on targets. The readout electronics of flexible force sensors require a voltage divider and an analog to digital converter, which are compact and simple [37]. Flexible force sensors have good sensitivity, take up less space and are not susceptible to strong magnetic fields, so they are a good choice to measure the capsule-tissue interaction force.

In this paper, we design a magnetically-guided capsule endoscope with flexible force sensors. Through these sensors, the interaction force that the internal permanent magnet applied on the intestinal wall is measured to avoid intestinal injury and the loss of magnetic coupling during colonoscopy. By analyzing the force measured by these sensors, we can recognize the motion status of the magnetic capsule, such as whether the capsule is following the motion of the external magnet. The paper is organized as follows: Section 2 presents the designed magnetic capsule with flexible force sensors and the calibration method of these sensors. Section 3 analyzes the force signal measured from flexible force sensors and proposes a control strategy to avoid the loss of magnetic coupling between the external and internal magnets. Section 4 presents the experiments that were performed to evaluate the proposed motion recognition and alignment methods. Section 5 summarizes the results and provides the conclusion of this study.

## 2. Mechanical Design

**Figure 2:**
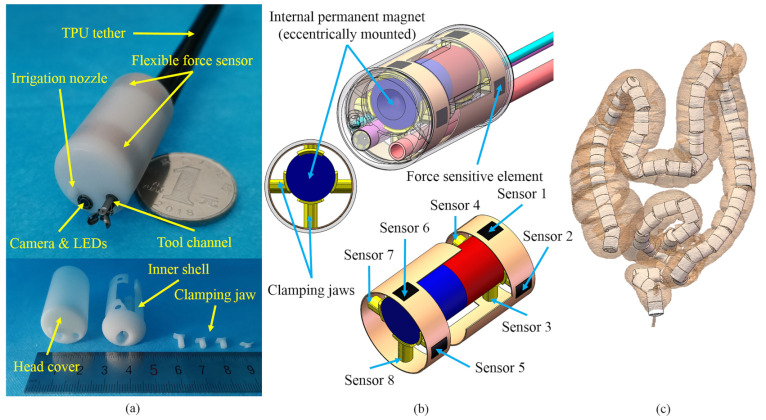
Designed magnetic capsule endoscope with flexible force sensors. (**a**) Mechanical structure. (**b**) Distribution of force sensitive elements. (**c**) Simulation of the motion of the designed magnetic capsule inside a reconstructed human large intestine.

The mechanical structure of the designed magnetic capsule endoscope is shown in Figure 2. Compared to electromagnetic solutions, permanent magnet is a more practical choice due to the reduction in cost, no need for large electrical currents and high strength-to-size ratio [29]. In this study, permanent magnets are chosen as the external and internal magnets for driving purposes.

The designed magnetic capsule is equipped with two flexible force sensors, an IPM, a nozzle for irrigation and insufflation, a camera with light-emitting diodes (LEDs), and an operating channel compatible with commercially-available biopsy instruments, as shown in Figure 2a. Electrical wires, as well as the tool channel and irrigation channel, are routed through a thermoplastic polyurethane (TPU) tether to connect with other equipment, such as readout electronics and air pump. When the large intestine is inflated, the magnetic capsule can more easily pass the folds of the large intestine and doctors can get a better view of the lining of the colon, so air inflation is necessary during magnetically-guided capsule colonoscopy. In this paper, the work environment of the magnetic capsule is an inflated large intestine. Two customized commercial flexible force sensors (ZQ01, RFP, China) are embedded in the inner shell and packaged by the head cover. Each flexible force sensor has four force sensitive elements (4 mm length and 3 mm width). The force range of the flexible force sensor is up to 5 N. Its response time is less than 10 ms, and its hysteresis error is ±4.5% of the full scale. The IPM is eccentrically mounted inside the capsule and fixed by eight clamping jaws, as shown in Figure 2b. The other side of the clamping jaw threads through the hole at the flank of the inner shell and touches the corresponding force sensitive element. Through clamping jaws, the magnetic force acting on the IPM is transmitted to force sensitive elements of flexible force sensors. Because the IPM is fixed inside the capsule endoscope, the magnetic force acting on the IPM through the EPM-IPM magnetic interaction is equal to the contact force between the IPM and the force sensitive elements if we ignore the influence of gravity. The interaction force of the magnetic capsule acting on the intestinal wall can also be regarded as the contact force between the IPM and the force sensitive elements if we ignore acceleration. Corresponding numbers of eight force sensitive elements are shown in Figure 2b, and serially numbered as Sensor 1 to 8 in this paper. Because the IPM is eccentrically mounted, the magnetic capsule tends to maintain the state that Sensor 1 and Sensor 6 are on top of the magnetic capsule during the intestine detection process. If the magnetic capsule rotates along the magnetization axis in some cases and the force value of Sensor 1 plus Sensor 6 is reduced to zero, we can rebuild the magnetic link between the IPM and EPM to repeat this state. The diameter and length of the capsule are 19 mm and 30 mm, respectively, and the diameter and length of the IPM (NdFeB, N52 grade) are 10 mm and 25 mm, respectively. We reconstruct 3D models of human large intestines from multiple-detector computed tomography (MDCT) data [38], and simulate the motion of the designed capsule inside these models, as shown in Figure 2c. The designed capsule can go through the lumen of the human large intestine, demonstrating that its dimensions are acceptable. 

**Figure 3:**
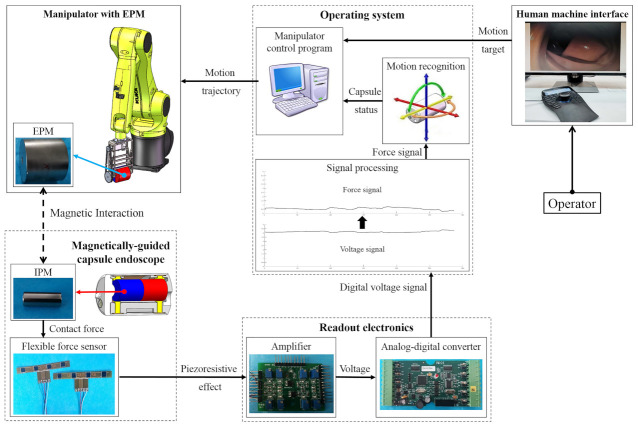
Hardware system of the designed magnetically-guided capsule endoscope.

The hardware system of the magnetically-guided capsule endoscope is presented in Figure 3. Through the human–machine interface, the operator inputs the desired motion trajectory of the capsule into the operating system installed on the computer. The operating system converts the motion trajectory into motion vectors and sends them to the controller of the manipulator (KR-C4, Kuka, Shanghai, China). The EPM is installed on the end of the manipulator and driven by it. The manipulator includes a serial manipulator (KR10-R1100, Kuka, Shanghai, China) and an auxiliary degree of freedom (DOF) which extends the workspace of the EPM and makes its workspace suitable for magnetically-guided capsule colonoscopy. After displacement of the EPM, due to the magnetic interaction between magnets, the magnetic force acting on the IPM is changed. Then, variations in the magnetic force are transmitted to force sensitive elements of the flexible force sensors through clamping jaws, changing the resistance of corresponding force sensitive elements. Their resistance value is converted to voltage signal by an amplifier (ZQ01, RFP, Hangzhou, China) and converted to digital signal by an analog-digital converter (5V measuring range, 12-bit resolution, 50 Hz sampling frequency; DAM3128, ARTTechnology, Beijing, China). After proper signal processing, it is possible to obtain the continuous flow of signals of the contact force that the IPM applies to force sensitive elements through the EPM-IPM magnetic interaction. By analyzing the changes of these signals, we can derive the current motion status of the magnetic capsule. When predetermined criterion is triggered, such as increased magnetic force tending to go beyond the limitation of the intestine wall, or decreased magnetic force tending to lose magnetic coupling between the EPM and IPM, the operating system issues a caution to the operator and corresponding control strategy is implemented to manage the problem. This can decrease the perforation risk of the magnetic capsule endoscope in clinical applications as well as operation difficulties for colonoscopists. 

**Figure 4:**
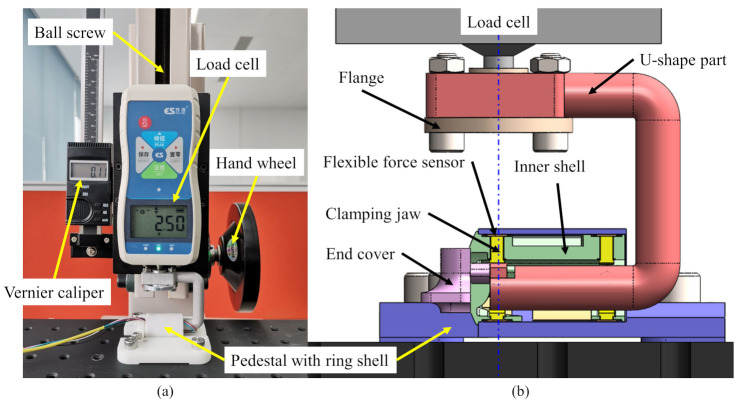
Sensor calibration. (**a**): The equipment for sensor calibration. (**b**): 3D model of the equipment.

**Figure 5:**
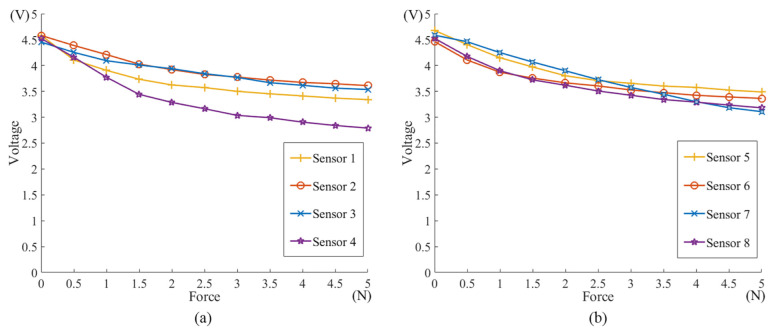
Measured voltage-force curves of each force sensitive element. (**a**): Voltage-force curves of Sensor 1 to 4. (**b**): Voltage-force curves of Sensor 5 to 8.

Before installing the flexible force sensor into the magnetic capsule endoscope, each force sensitive element was calibrated. An equipment is properly designed to calibrate the flexible force sensor as shown in Figure 4a, and corresponding 3D model (SolidWorks, Dassault Systemes, Waltham, MA, USA) is shown in Figure 4b. Flexible force sensors are installed on the inner wall in the same way as the designed magnetic capsule endoscope. They are then inserted into the ring shell of the pedestal together and fixed by the end cover. One side of the clamping jaw clings to the force sensitive element and the other side touches the U-shaped part. The U-shaped part is mounted on the end of the load cell (SH-100, Nscing, Nanjing, China) by the flange. By turning the hand wheel, the ball screw translates the load cell with the U-shaped part, which applies force to the clamping jaw. Through the U-shaped part, the force applied on the force sensitive element can be transmitted to the end of the load cell. Thus, we can control the contact force acting on the force sensitive element and read the force value through the load cell. Corresponding voltage value is input into the computer and can be read through a software (Matlab, Math Works, Natick, MA, USA). In this way, we measure voltage values of each force sensitive element under various forces and elaborate their voltage-force curves, as shown in Figure 5. In practice, we measure voltage values of each force sensitive element, and interpolate each voltage value into the piecewise linear formula of corresponding voltage-force curve to calculate the corresponding force acting on each force sensitive element.

## 3. Motion Recognition of the Magnetically-Guided Capsule

**Figure 6:**
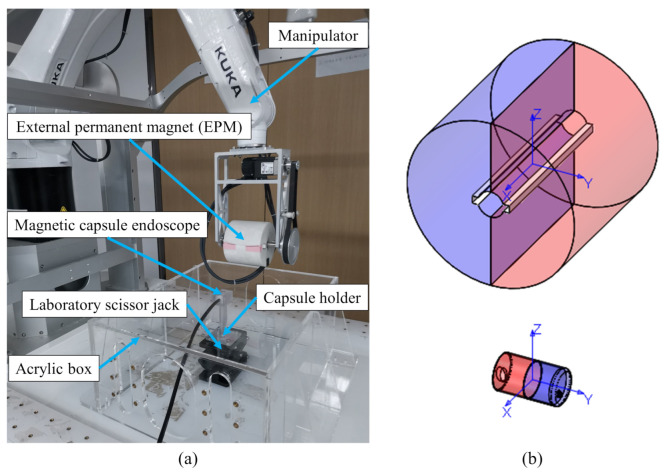
The platform used to recognize the motion status of the magnetic capsule endoscope. (**a**): Experiment platform. (**b**): Local coordinate systems of the EPM and the magnetic capsule.

The human large intestine is composed of teniae coli, haustra, and epiploic appendages [39]. Contractions of the teniae coli bunch up the colon into a succession of pouches called haustra, which are responsible for the wrinkled appearance of the colon. During the magnetically-guided capsule colonoscopy, the magnetic capsule may fall into the haustra, restricting its motion. In addition, the human large intestine is surrounded by other organs and has complex morphologic features [40]. The motion of the magnetic capsule can also be restricted due to the pressure of surrounding organs. Because the magnetic capsule is driven by the EPM, its motion direction is already known, and we need to recognize whether the magnetic capsule is following the motion of the EPM or is restricted by human organs. By analyzing the force signals measured from the flexible force sensors, we can measure the interaction force between the magnetic capsule and surrounding organs and recognize the motion status of the magnetic capsule. A platform including the manipulator with an auxiliary DOF and the EPM is used to drive the magnetic capsule endoscope and distinguish its motion status, as shown in Figure 6a. Additionally, to simulate the situation that the motion of the magnetic capsule is restricted, a capsule holder is fabricated and installed on a laboratory scissor jack. The capsule holder can be lifted by the scissor jack to fix the magnetic capsule with the acrylic box. The motions of the EPM and the magnetic capsule are, respectively, described under their local coordinate systems. The local coordinate system is fixed on object’s geometric center, and its direction is shown in Figure 6b. The Y-axis is always parallel to the magnetization direction of the magnet. In this paper, we want to recognize whether the magnetic capsule can follow the motion of the EPM when the EPM moves in various directions. Variations in the force signal caused by motions of the EPM are analyzed and discussed below. 

### 3.1. EPM Translating along Its Z-Axis

When the EPM moves downward, it applies larger force on the IPM and drags the IPM upward. Therefore, when the Z-axis of the EPM and the IPM is collinear and the EPM translates along its negative Z-axis, the motion direction of the magnetic capsule is the positive Z-axis, which is opposite to the EPM. To stably control the magnetic capsule, we make the capsule always cling to the intestinal inner wall during the colonoscopy. In our previous work, we measured the perforation force of the porcine large intestine and suggested that magnetic capsule’s interaction force applied to the intestinal wall by the magnetic capsule should be below 10 N [41]. Considering the safety of the magnetically-guided capsule colonoscopy and the reduction in discomfort and pain for patients, we set the allowed maximum force of each force sensitive element at 3 N, and the force of Sensor 1 plus Sensor 6, which are always on top of the capsule and touching the intestine wall, at below 5 N. In addition, to prevent the loss of the magnetic coupling, the allowed minimum force of Sensor 1 plus Sensor 6 is set at 0.5 N. Thus, the criterion to recognize whether the magnetic capsule can follow the motion of the EPM along the Z-axis can be defined as:

(1) $$F_{i}\left(t\right)<3,\left(i=1,2,...,6\right)$$

(2) $$0.5<F_{1}\left(t\right)+F_{6}\left(t\right)<5$$

where *t* is time, and $F_{i}\left(t\right)$, (i = 1, 2, …, 6) are the force values measured by Sensor 1 to Sensor 6. If the force is beyond the threshold, the EPM needs to be moved upward to decrease the attractive force acting on the IPM. If the force is always below the threshold, we need realign the EPM and the IPM or move the EPM downward when the position of the EPM is too high.

### 3.2. EPM Translating along Its X-Axis

**Figure 7:**
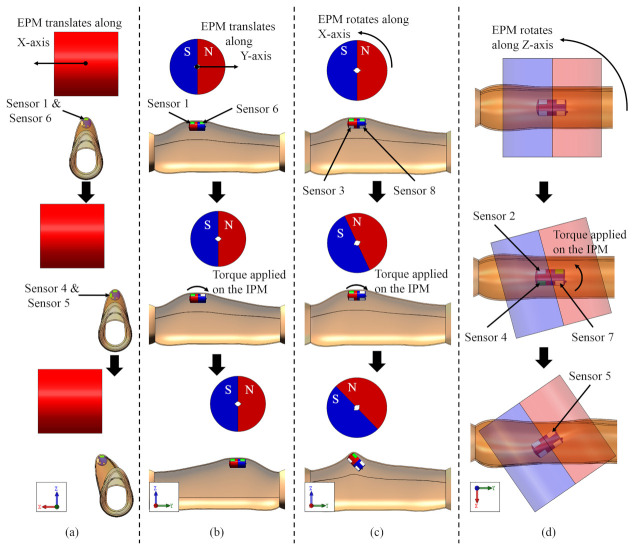
Corresponding motion directions of the magnetic capsule when the EPM moves in various directions. (**a**) The EPM translates along its X-axis. (**b**) The EPM translates along its Y-axis. (**c**) The EPM rotates along its X-axis. (**d**): The EPM rotates along its Z-axis.

**Figure 8:**
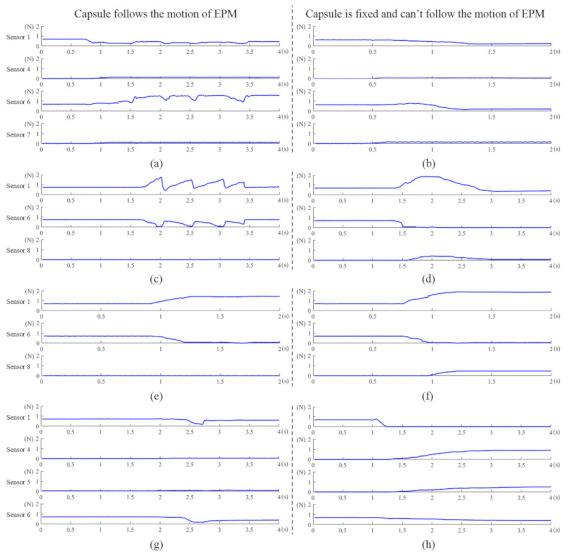
When the EPM moves along various directions, corresponding force signals are measured by force sensitive elements. The abscissa axis of each curve indicates time and the ordinate axis indicates force. (**a**) The EPM translates along its positive X-axis and the magnetic capsule follows its motion. (**b**) The EPM translates along its positive X-axis and the magnetic capsule can’t follow its motion. (**c**) The EPM translates along its positive Y-axis and the magnetic capsule follows its motion. (**d**) The EPM translates along its positive Y-axis and the magnetic capsule can’t follow its motion. (**e**) The EPM rotates along its positive X-axis and the magnetic capsule follows its motion. (**f**) The EPM rotates along its positive X-axis and the magnetic capsule can’t follow its motion. (**g**) The EPM rotates along its positive Z-axis and the magnetic capsule follows its motion. (**h**) The EPM rotates along its positive Z-axis and the magnetic capsule can’t follow its motion.

Using the platform shown in Figure 6a, we simulate the situation that the magnetic capsule can and cannot follow the motion of the EPM. Figure 7 presents motion directions of the magnetic capsule when the EPM moves along various directions. Force signals of each situation are recorded and shown in Figure 8a. In Figure 8, to save space, we just draw signal curves of sensors with obvious changes.

When the EPM translates along its X-axis, the IPM tends to follow the motion of the EPM and applies force on the Sensor 4 and Sensor 7, as shown in Figure 7a. At the moment, forces applied on the Sensor 1 and Sensor 6 decrease because the distance between the EPM and the IPM increases. Then, if the drag force applied on the IPM is beyond the resistance, the EPM and the IPM are realigned and forces applied on the Sensor 1 and Sensor 6 increase because the distance between the EPM and the IPM decreases. So if the magnetic capsule can follow the motion of the EPM along the X-axis, the force curve of the Sensor 1 or Sensor 6 has a local minimum point, as shown in Figure 8a. If the motion of the magnetic capsule is restricted and it cannot follow the motion of the EPM, forces applied on the Sensor 1 and Sensor 6 continually decrease because the distance between the EPM and the IPM continually increases. In this case, force curves of the Sensor 1 and Sensor 2 are smooth and tend to zero, as shown in Figure 8b. Thus, the criterion to recognize that the magnetic capsule follows the motion of the EPM along the X-axis can be defined as:

(3) $$\left\{\begin{array}{c} \left[F_{i}\left(t+\Delta t\right)-F_{i}\left(t\right)\right]\;\times \;\left[F_{i}\left(t-\Delta t\right)-F_{i}\left(t\right)\right]\;\geq \;0\\ \left[F_{i}\left(t+\Delta t\right)-F_{i}\left(t\right)\right]\;+\;\left[F_{i}\left(t-\Delta t\right)-F_{i}\left(t\right)\right]\;>\;\delta_{1},\left(i=1\;or\;6\right) \end{array}\right.$$

where Δt is an increment of time, and $\delta_{1},\delta_{2},\delta_{3},\delta_{4}$ are the preset thresholds.

### 3.3. EPM Translating along Its Y-Axis

When the EPM translates along its Y-axis, it applies torque on the IPM, as shown in Figure 7b. In this case, the force applied on the Sensor 1 increases and the force applied on the Sensor 6 decreases. Then, if the drag force applied on the IPM is beyond the resistance, the EPM and the IPM are realigned. In this case, the force applied on the Sensor 1 decreases and the force applied on the Sensor 6 increases. So if the magnetic capsule can follow the motion of the EPM along the Y-axis, the force curve of the Sensor 1 or Sensor 6 has a local minimum point, as shown in Figure 8c. If the motion of the magnetic capsule is restricted, forces applied on the Sensor 1 and Sensor 6 continually decrease because the distance between the EPM and the IPM continually increases. In this case, force curves of the Sensor 1 and Sensor 2 are smooth and tend to zero, as shown in Figure 8d. Thus, its recognition formula is the same as Equation (3):

(4) $$\left\{\begin{array}{c} \left[F_{i}\left(t+\Delta t\right)-F_{i}\left(t\right)\right]\;\times \;\left[F_{i}\left(t-\Delta t\right)-F_{i}\left(t\right)\right]\;\geq \;0\\ \left[F_{i}\left(t+\Delta t\right)-F_{i}\left(t\right)\right]\;+\;\left[F_{i}\left(t-\Delta t\right)-F_{i}\left(t\right)\right]\;>\;\delta_{2},\left(i=1\;or\;6\right) \end{array}\right.$$

When the EPM moves along the positive Y-axis, the force measured by the Sensor 1 is used as the recognition criterion, and when the EPM moves along the negative Y-axis, the force measured by the Sensor 6 is used as the recognition criterion. Equations (3) and (4) can also be regarded as the criterion to recognize that the magnetic capsule can follow the motion of the EPM in the X-Y plane.

### 3.4. EPM Rotating along Its X-Axis

When the EPM rotates along its X-axis, it applies a torque on the IPM, and the rotation direction of the magnetic capsule is opposite to the EPM, as shown in Figure 7c. In this case, the force applied on the Sensor 1 increases and the force applied on the Sensor 6 decreases. Then, if the magnetic capsule can follow the rotation of the EPM along the X-axis, forces applied on the Sensor 3 and Sensor 8 tend to zero, as shown in Figure 8e. If the rotation of the magnetic capsule is restricted, the repulsive force that the EPM applies on the IPM increases. This repulsive force is transferred to the Sensor 3 or Sensor 8 and their values increase, as shown in Figure 8f. The rotation range of the EPM along the X-axis is limited within ±45°. Thus, the criterion to recognize that the magnetic capsule follows the rotation of the EPM along the X-axis can be defined as:

(5) $$\left\{\begin{array}{c} F_{3}\left(t\right)\leq \delta_{3}\\ F_{8}\left(t\right)\leq \delta_{3} \end{array}\right.$$

### 3.5. EPM Rotating along Its Z-Axis

When the EPM rotates along the Z-axis, it applies torque on the IPM, as shown in Figure 7d. Force values of the Sensor 4 and Sensor 5 increase when the EPM rotates along the positive Z-axis, and force values of the Sensor 2 and Sensor 7 increase when the EPM rotates along the negative Z-axis. Then, if the torque applied on the IPM is beyond the resistance, the EPM and the IPM are realigned, and forces applied on the Sensor 4 and Sensor 5 tend to zero, as shown in Figure 8g. If the rotation of the magnetic capsule is restricted, the torque that the EPM applies on the IPM increases, and forces applied on the Sensor 4 and Sensor 5 increase, as shown in Figure 8h. Thus, the criterion to recognize that the magnetic capsule follows the rotation of the EPM along the Z-axis can be defined as:

(6) $$\left\{\begin{array}{c} F_{4}\left(t\right)+F_{5}\left(t\right)\leq \delta_{4}\\ F_{2}\left(t\right)+F_{7}\left(t\right)\leq \delta_{4} \end{array}\right.$$

Using Equation (1) to Equation (6), we can reliably and quickly recognize the motion status of the magnetic capsule endoscope, including whether the force applied on the intestinal wall is too large, the magnetic link is effective, and the magnetic capsule is restricted by human organs. These interaction information is important for control decisions of the operator and the safety of the patient.

### 3.6. Realigning the EPM and the IPM

**Figure 9:**
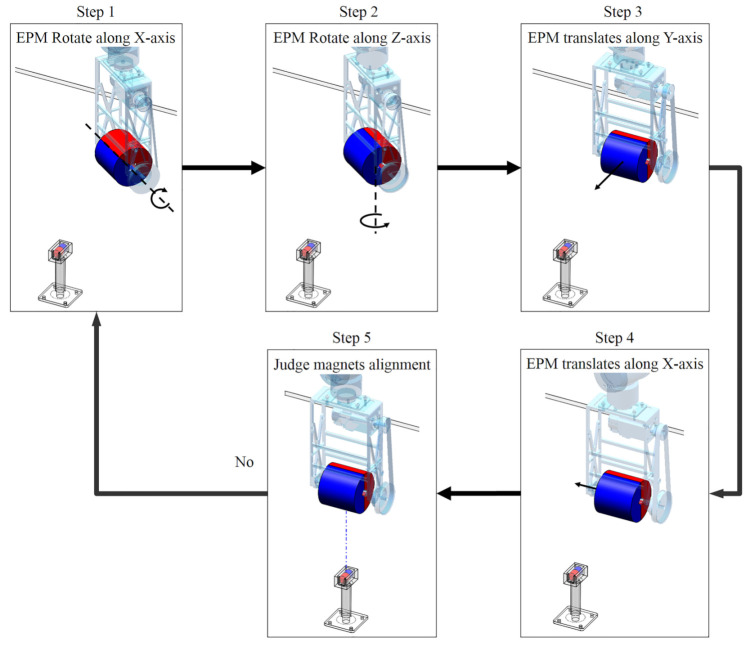
Control method used to realign the EPM and the IPM to prevent loss of the magnetic coupling.

One feature of the magnetic based actuation is the asynchronous motion of the EPM and the IPM. The IPM starts to follow the motion of the EPM after the EPM has moved a distance. If the EPM moves too far, the magnetic force link between the EPM and the IPM may break and the magnetic capsule gets out of control. To prevent this situation, we need to monitor the force value of the Sensor 1 plus Sensor 6 which can be regarded as the magnetic force exerted by the IPM along its Z-axis in real-time. If this force is below the preset threshold, the EPM and the IPM need to be realigned. Moreover, due to line-of-sight limitations, the initial location of the magnetic capsule is unknown in clinical applications. In this case, we can move the EPM close to the possible location of the magnetic capsule until the force value of the Sensor 1 plus Sensor 6 is within the preset threshold, and then align the EPM and the IPM. Proposed alignment method can be divided into five steps, as shown in Figure 9.

**Figure 10:**
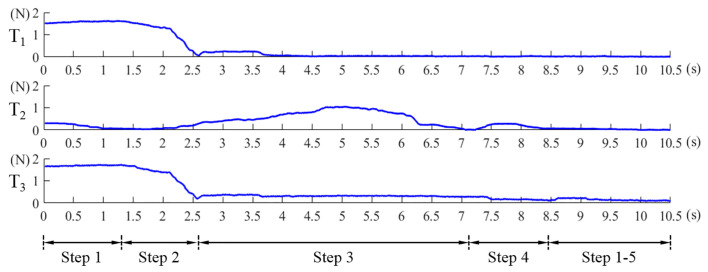
Values of $T_{1}$, $T_{2}$ and $T_{3}$ in Equations (7)–(9). The abscissa axis of each curve indicates time and the ordinate axis indicates force.

Step 1. rotate the EPM along its X-axis until the Z-axis of the EPM is vertical to the ground.
Step 2. rotate the EPM along its Z-axis direction which decreases the value of $\left|F_{2}\left(t\right)+F_{7}\left(t\right)-F_{4}\left(t\right)-F_{5}\left(t\right)\right|$ until:
  (7) $$T_{1}\;=\;\left|F_{2}\left(t\right)+F_{7}\left(t\right)-F_{4}\left(t\right)-F_{5}\left(t\right)\right|\leq \eta_{1}$$
Step 3. translate the EPM along its Y-axis direction which decreases the value of $\left|F_{1}\left(t\right)-F_{6}\left(t\right))\right|$ until:
  (8) $$T_{2}\;=\;\left|F_{1}\left(t\right)-F_{6}\left(t\right)\right|\leq \eta_{2}$$
Step 4. translate the EPM along its X-axis direction which decreases the value of $F_{2}\left(t\right)+F_{4}\left(t\right)+F_{5}\left(t\right)+F_{7}\left(t\right)$ until:
  (9) $$T_{2}=F_{2}\left(t\right)+F_{4}\left(t\right)+F_{5}\left(t\right)+F_{7}\left(t\right)\leq \eta_{3}$$
Step 5. repeat the Step 1 to Step 4 until the Equations (8) and (9) are simultaneously satisfied. $\eta_{1}$, $\eta_{2}$, $\eta_{3}$ are the preset thresholds. Values of Equation (7), Equations (8) and (9) in this alignment process are shown in Figure 10. The abscissa axis of each curve indicates the value of time and the ordinate axis indicates the value of force. If the magnetic link between the EPM and the IPM has already been broken or is not established, such as at the beginning of the magnetic capsule colonoscopy, the EPM can be moved close to the possible position of the magnetic capsule to establish magnetic link. Once the force value of the Sensor 1 plus Sensor 6 is within the preset threshold, we can implement the alignment method to align the EPM and the IPM. The alignment method based on flexible force sensors is suitable for most situation of the magnetic capsule colonoscopy and less likely to be affected by the interference compared with other localization strategies.

**Figure 11:**
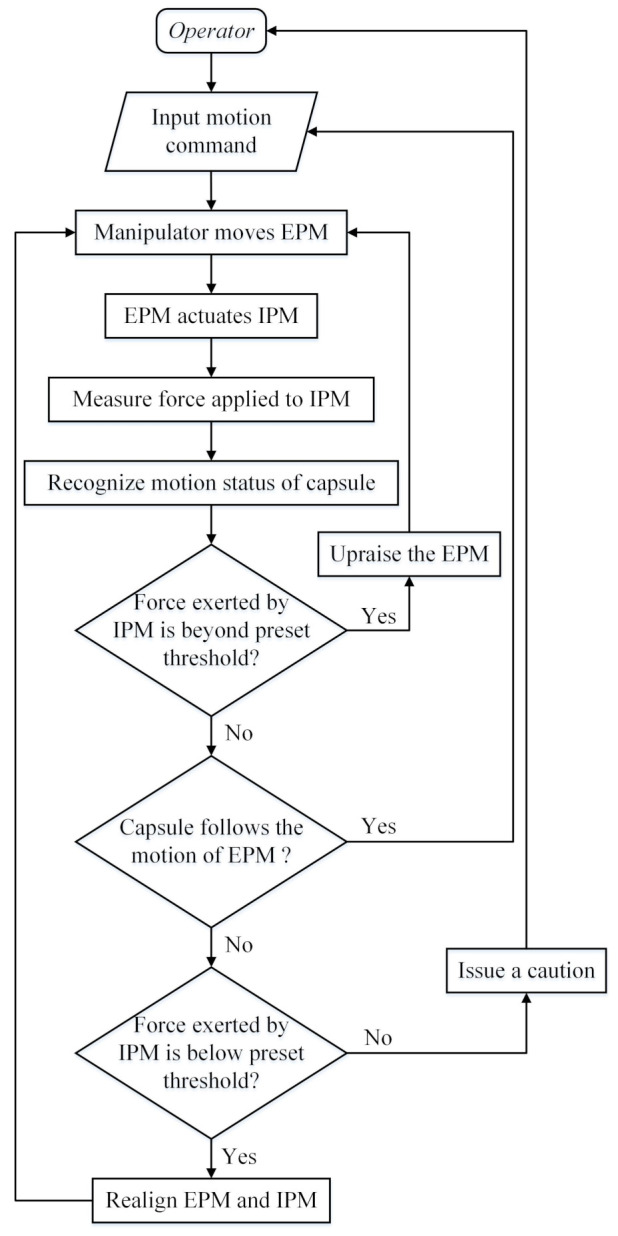
The control flow chart of the designed magnetic capsule endoscope system.

A flow chart of the control strategy for the designed magnetic capsule endoscope system is shown in Figure 11. After the operator input motion command into the manipulator controller, the manipulator moves the EPM. Then, the magnetic force applied to the IPM is changed and the EPM asynchronous actuates the IPM. Through the force sensitive element, the operating system measures the contact force applied to force sensitive elements by the IPM and recognizes the motion status of the magnetic capsule. This recognition method is only executed when the EPM is moving. First, to avoid injuring the intestinal tissue, the operating system evaluates whether the contact force exerted by the IPM is beyond the preset threshold. If the result is “YES”, the manipulator raises the EPM to reduce the contact force applied on the intestine wall. Then the operating system evaluates whether the magnetic capsule is following the motion of the EPM. If the result is “YES”, the operator can continue inputting command. If the result is “NO”, the operating system needs to evaluate whether the contact force exerted by the IPM is below the preset threshold. If this force value is within the threshold, the operating system issues a caution to the operator declaring that the magnetic capsule is not following the motion of the EPM and waits for further command. If the contact force exerted by the IPM is below the preset threshold, the magnetic force link between the EPM and the IPM will tend to break, and the proposed alignment method will need to be implemented. This control strategy ensures controllability of the magnetic capsule endoscope during the colonoscopy and is important for the medical device.

## 4. Experimental Validation

**Figure 12:**
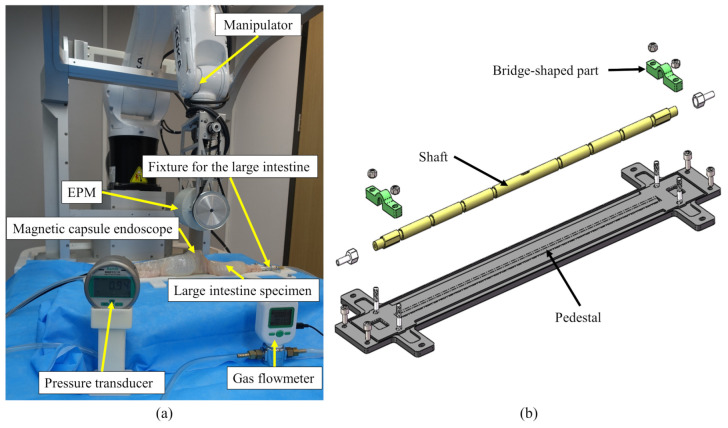
The platform used to evaluate the proposed motion recognition method of the magnetic capsule endoscope. (**a**): Experiment platform. (**b**): The fixture designed for mounting the large intestine.

To evaluate the proposed recognition method, we performed Ex Vivo tests on a properly designed experiment platform. The situation that the magnetic capsule is restricted from translating is often caused by the wrinkled structure of the haustra. For it was the most common event in our previous tests, the tests were performed under the condition that the EPM translates along its Y-axis. The platform mainly consists of a manipulator, a fixture for mounting the large intestine, an EPM, the designed magnetic capsule endoscope and the large intestine specimen, as shown in Figure 12a. The fixture for mounting the large intestine includes a shaft which goes through the intestinal lumen and is mounted on the pedestal by two bridge shaped parts at both sides, as shown in Figure 12b. The shaft is hollow, and a through hole is drilled in the middle of the shaft so that it is possible to inflate the large intestine with an air pump connected to one side of the shaft. To fix the specimen, we tightened the large intestine at the groove of the shaft with ribbons. The porcine large intestine was chosen as the specimen in this test because it has similar mechanical properties to human tissues [42]. The specimen was removed from breeding pig that had been processed from the food chain immediately following culling and cleaning, so ethical approval is not required for this experiment. 

After obtaining the flow signal of the force sensor, we had to preprocess the raw time-series force data. The raw force data were filtered using a one order low pass filter and divided into 0.4 s segment using a window size of 20 with 10 samples overlapping between consecutive windows. The sampling frequency of the flexible force sensor is 50 Hz. The experiment in which the magnetic capsule followed the motion of the EPM along its Y-axis was performed 30 times, and the experiment in which the magnetic capsule could not follow the motion of the EPM was performed 20 times. Corresponding force data were recorded. A total of 20 sets of force data curves measured under the condition that the magnetic capsule followed the motion of the EPM were analyzed to determine the threshold $\delta_{2}$ as training set. Other force data were regarded as test set. By choosing suitable threshold, 47 sets of force data were correctly classified, especially all data measured under the condition that the magnetic capsule could not follow the motion of the EPM were correctly classified. For thresholds $\delta_{1}$, $\delta_{3}$ and $\delta_{4}$, corresponding experiments need to be performed in situations that the magnetic capsule is restricted from translating along the X-axis, and rotating along the X-axis and Z-axis. These situations are often caused by the pressure of other organs surrounding the large intestine and ca not be simulated in an test. In future work, we need further tests to determine the values of these thresholds.

**Figure 13:**
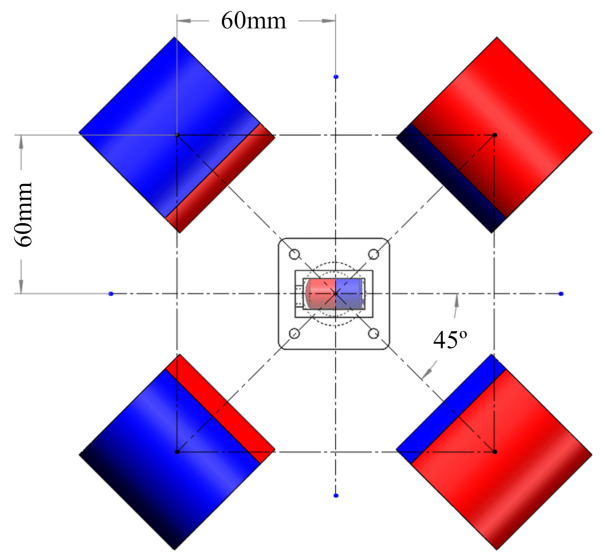
Relative positions of the EPM and the IPM in error analysis experiments. Experiments were performed to select appropriate thresholds for proposed alignment method.

**Figure 14:**
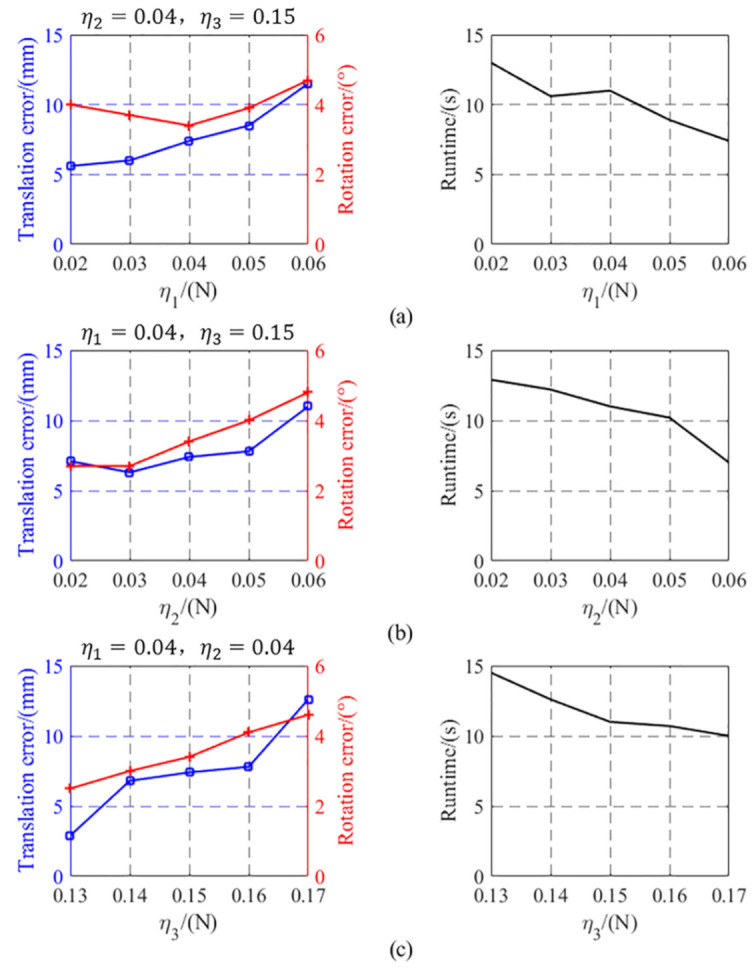
Translational error, rotational error, and runtime of each error analysis experiment. (**a**): $\eta_{2}=0.04$, $\eta_{3}=0.15$ and $\eta_{1}$ is the variable. (**b**): $\eta_{1}=0.04$, $\eta_{3}=0.15$ and $\eta_{2}$ is the variable. (**c**): $\eta_{1}=0.04$, $\eta_{2}=0.04$ and $\eta_{3}$ is the variable.

For the alignment method, a series of experiments were performed to select the appropriate values of $\eta_{1}$, $\eta_{2}$, $\eta_{3}$, Corresponding experiment platform is shown in Figure 6a. The magnetic capsule with a known position was fixed in these experiments and the EPM was placed in four different positions, as shown in Figure 13. For each $\eta_{1}$, $\eta_{2}$, $\eta_{3}$, we performed four groups of experiments and each group was performed four times. The angle of the EPM rotation along its X-axis was set as ±45°. Selected postures of the EPM can represent most situations during a colonoscopy. In each experiment, the translational error ($E_{T}$) and rotational error ($E_{R}$) between the EPM and the magnetic capsule were recorded. The runtime for realigning the EPM and the IPM was also recorded and averaged. Considering the length of the EPM ($L_{EPM}$) and the diameter of the IPM ($D_{IPM}$), the translational error is calculated as follows:

(10) $$E_{T}=\frac{1}{16}\sum_{i=1}^{16}\sqrt{{\left(E_{TYi}\right)}^{2}+{\left(max\left\{E_{TXi}-L_{EPM}/2-D_{IPM}/2,\;0\right\}\right)}^{2}}$$

(11) $$E_{R}=\frac{1}{16}\sum_{i=1}^{16}E_{RZi}$$

where $E_{TXi}$ and $E_{TYi}$ are relative distances between the EPM and the IPM along the X-axis and Y-axis of the magnetic capsule, respectively, and $E_{RZi}$ is the relative rotation angle between the EPM and the IPM along the Z-axis of the magnetic capsule in the ith(i=1…16) experiment. Error value and runtime of each $\eta_{1}$, $\eta_{2}$, $\eta_{3}$ are shown in Figure 14. In Figure 14a, the rotational error increases after $\eta_{1}$ less than 0.04, so we set $\eta_{1}=0.04$. In Figure 14b, to get less position error, we set $\eta_{2}=0.03$. In Figure 14c, to reduce the runtime, we set $\eta_{3}=0.15$. Using selected thresholds, the translational error of proposed alignment method was 7.04 ± 0.71 mm, the rotational error was 3.13 ± 0.47° and runtime was 11.4 ± 0.39 s. From the results of these experiments, we can that see proposed motion recognition and alignment methods are effective.

## 5. Conclusions

This research analyzes the interaction force between the magnetic capsule and its surroundings with the aim of ensuring the safety and controllability of the magnetic capsule endoscope during the large intestine detection process. A magnetic capsule with flexible force sensors is designed to measure the interaction force between the magnetic capsule and the intestinal wall. By controlling the capsule-tissue interaction force within a preset threshold, we can avoid causing the patient discomfort and losing the magnetic coupling between the EPM and the IPM. In addition, due to the complex structure of the human large intestine and asynchronous motion of the EPM and the IPM, the maneuverability of the magnetic capsule is inevitably restricted during a colonoscopy. By analyzing force signals of force sensitive elements, we recognize the motion status of the magnetic capsule, and corresponding formulas are given to classify whether the magnetic capsule is following the motion of the EPM. The recognition accuracy in Ex Vivo tests can reach 94% when the EPM translates along its Y-axis. A control method is proposed to realign the EPM and the IPM before the loss of their magnetic coupling. Its translational error, rotational error, and runtime are 7.04 ± 0.71 mm, 3.13 ± 0.47°, and 11.4 ± 0.39 s, respectively. Based on the recognition and alignment methods, a control strategy is proposed to prevent the magnetic capsule from losing control during the magnetically-guided capsule colonoscopy.

## Figures and Tables

**Table 1:** Comparison of current magnetically-guided capsule colonoscopes.

Researcher	Actuation Strategy	IPM Size (mm)	Capsule Size (mm)	Control Method	Sensor Type	Ref
Ciuti et al. 2010 VECTOR EUR project	Permanent magnet	3.2 × 19.1 (3 magnets)	13.5 × 29.5	Position control	Hall effect sensor & IMU	[14,18]
Lucarini et al. 2015 SUPCAM EUR project	Electromagnet	11.4 × 5.5	37 × 37	Force control	Current of electromagnet	[21]
Bianchi et al. 2017 ENDOO EUR project	Permanent magnet	-	-	Position control	Hall effect sensor & IMU	[17,22]
Nouda et al. 2018	Electromagnet	-	11 × 45	Silicone fin with magnet	-	[23]
Taddese et al. 2019	Permanent magnet	11.1 × 11.1	20 × 22	Force control	Hall effect sensor & IMU	[19,24]
Norton et al. 2019	Permanent magnet	11.1 × 11.1	21 × 39	Position control	Micro ultrasound transducer	[20]

## Data Availability

Data sharing not applicable.

## Abbreviations

The following abbreviations are used in this manuscript:

IPM	Internal permanent magnet
EPM	External permanent magnet
CRC	Colorectal cancer
IMU	Inertial measurement unit
EUR	European
LEDs	Light-emitting diodes
TPU	Thermoplastic polyurethane
MDCT	Multiple-detector computed tomography
DOF	Degree of freedom
